# Ravulizumab for relapse prevention in AQP4-IgG–positive neuromyelitis optica spectrum disorder: a 2-year follow-up case report

**DOI:** 10.3389/fmed.2026.1827687

**Published:** 2026-06-12

**Authors:** Nabil Akkawi, Elkhansa Hassabo Mohamed, Ahmed Shatila

**Affiliations:** 1 Neurology, HMS Mirdif Hospital, Dubai, United Arab Emirates; 2 Neurology, Sheikh Shakhbout Medical City, Abu Dhabi, United Arab Emirates

**Keywords:** aquaporin-4, complement C5 inhibitor, disease-modifying treatment, neuromyelitis optica, ravulizumab

## Abstract

**Background:**

Neuromyelitis optica spectrum disorder (NMOSD) is a rare autoimmune disease of the central nervous system, distinct from multiple sclerosis, characterized by severe inflammatory attacks targeting the optic nerves, spinal cord, and brainstem. Treatment has evolved from broad immunosuppressants to targeted biologics, such as complement inhibitors (ravulizumab/eculizumab), supported by strong evidence, including the CHAMPION-NMOSD trial for ravulizumab in AQP4-Ab+ve NMOSD patients. This case highlights the importance of prompt diagnosis and innovative treatment approaches in the management of NMOSD.

**Case presentation:**

A 20-year-old woman presented with acute brainstem dysfunction (dysarthria, diplopia) and a 1-month history of area postrema syndrome (APS), progressing to dysphagia and dyspnea requiring ICU admission. Diagnostic workup revealed AQP4-IgG+ NMOSD with characteristic lesions in the area postrema, pons, and cervical cord (C1–C3). The patient achieved complete clinical recovery with ravulizumab, with near-complete radiological resolution at 3 months and clinical and radiological stability through ~24 months of follow-up, highlighting the efficacy of post-acute initiation of complement inhibition in severe NMOSD.

**Conclusion:**

This case demonstrates the diagnostic and therapeutic challenges of AQP4-IgG+ NMOSD in a young patient presenting with area postrema syndrome. The patient remained clinically stable and free of new MRI activity through ~24 months of follow-up on ravulizumab.

## 1 Introduction

Neuromyelitis optica spectrum disorder (NMOSD) is a rare autoimmune condition of the central nervous system that primarily affects the optic nerves, spinal cord, and brainstem (1). The identification of aquaporin-4 immunoglobulin G autoantibodies (AQP4-IgG) in 2004 transformed our understanding of this disease, distinguishing it from multiple sclerosis (2). These autoantibodies target water channels on astrocytes, triggering complement-mediated inflammation that leads to characteristic magnetic resonance imaging (MRI) lesions (3). The 2015 International Consensus Diagnostic Criteria established clear guidelines for diagnosis, emphasizing the importance of antibody testing and specific imaging findings (1).

Treatment approaches have evolved significantly in recent years. While traditional therapies relied on broad immunosuppressants like rituximab and azathioprine, the 2024 German Neuromyelitis Optica Study Group (NEMOS) guidelines recommend targeted biologics as first-line treatment (4). These include complement inhibitors such as ravulizumab and eculizumab, B-cell therapies such as inebilizumab, and interleukin-6 (IL-6) receptor blockers such as satralizumab (5, 6). The CHAMPION-NMOSD trial demonstrated ravulizumab's efficacy in AQP4-IgG-positive NMOSD, with a 98.6% reduction in relapse risk (HR 0.014; *p* < 0.001) (7), while case reports have shown its effectiveness in refractory cases (8) and for chronic symptoms (9).

In the United Arab Emirates (UAE), NMOSD presents unique challenges with a prevalence of 1.76 per 100,000 and 56%−65% AQP4-IgG seropositivity (10). Diagnostic delays are common due to limited access to specialized testing (11). While high-dose steroids remain widely used (94% of cases), newer biologics face significant barriers, including high costs (approximately $500,000 annually). Our decision to use ravulizumab as first-line therapy reflects current evidence showing that early, targeted intervention can prevent the disability seen in 58% of untreated patients within 1 year (12). This approach represents a shift toward precision medicine in NMOSD management, though challenges remain in ensuring equitable access to these advanced therapies.

## 2 Case presentation

A 20-year-old woman presented with acute-onset diplopia, blurred vision, and dysarthria progressing over 24 h, accompanied by distal paresthesia in her feet and a sensation of tongue heaviness. These neurological symptoms were preceded by a 1-month history of persistent dizziness with photophobia, nausea, and frequent vomiting episodes that had been initially attributed to gastritis, diagnosed via endoscopy at an outside facility. Her medical history was notable for a first-degree cousin with multiple sclerosis.

The patient was conscious and fully oriented to time, place, and person, with stable vital signs. Systemic examination was unremarkable except for neurological findings. She exhibited cerebellar ataxia with a leftward fall tendency and retropulsion, accompanied by bilateral dysmetria and rotatory nystagmus. Cranial nerve involvement included blurred vision due to palatal paralysis, dysarthria, and dysphagia. Motor examination revealed mild symmetrical weakness (4/5) in both proximal and distal muscle groups of the upper and lower limbs. The cough reflex remained intact. The expanded disability status scale (EDSS) was 5.0.

During the first 24 h of hospitalization, she developed new-onset dyspnea necessitating intensive care unit (ICU) transfer. Her condition continued to decline, with worsening dysphagia and dysarthria (EDSS 8.0). Initial laboratory workup, including complete blood count, C-reactive protein, urinalysis, electrolytes, and liver and renal function tests, showed no abnormalities. Brain MRI demonstrated multiple T2-hyperintense lesions with gadolinium enhancement involving the pons and dorsal medulla (particularly the area postrema; Figure 1). Spinal MRI revealed a longitudinally extensive lesion extending from C1 to C3. Quantitative ophthalmological assessment was performed using visual evoked potentials and Bedside confrontation visual field (CVF) testing; however, formal automated perimetry was not available. Visual evoked potential testing showed delayed latencies for the P100, N75, and N145 waves bilaterally, indicating optic pathway involvement (Figure 2). CVF demonstrated bilateral generalized reduction in visual field sensitivity.

**Figure 1:**
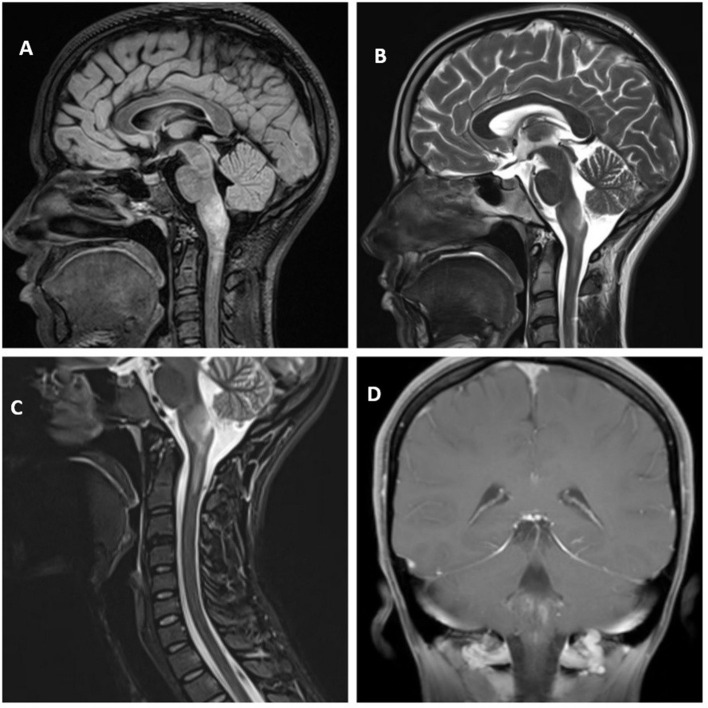
Brain and Cervical Spinal Cord MRI Findings. **(A)** Sagittal FLAIR brain MRI and **(B)** Sagittal T2-weighted MRI: Hyperintense lesion involving the dorsal brainstem (medulla/area postrema region) with extension toward the cervicomedullary junction. **(C)** Sagittal T2-weighted cervical spine MRI: Longitudinally extensive T2 hyperintense lesion (LETM) extending from the cervicomedullary junction down multiple cervical segments with central cord predominance. **(D)** Coronal post-contrast T1-weighted brain MRI: Patchy contrast enhancement in the brainstem region suggestive of active inflammation and blood–brain barrier disruption.

**Figure 2:**
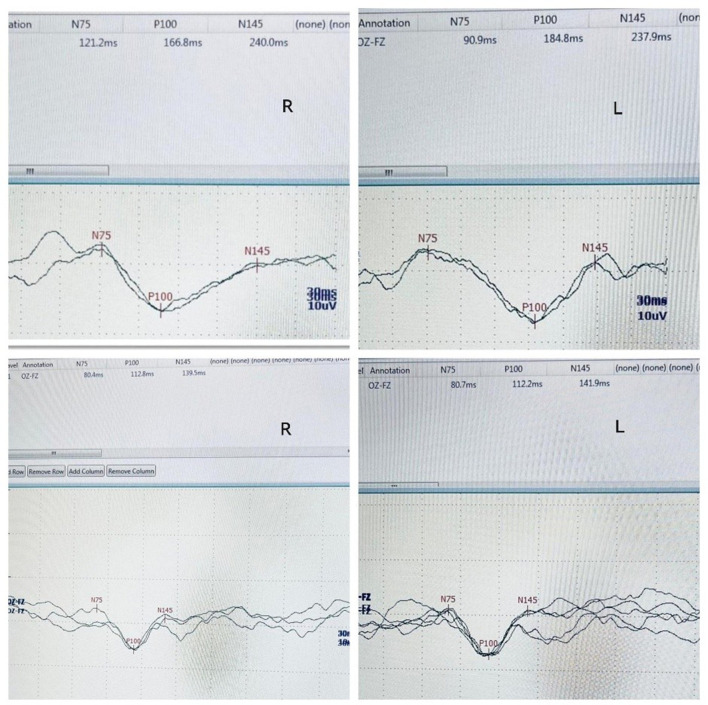
Visual Evoked Potentials (VEP) before and after 3 months treatment: **(Top Panels)**: Pre-treatment VEP (Right and Left Eyes): Prolonged P100 latencies bilaterally, Right eye: ~166.8 ms, Left eye: ~184.8 ms, reduced conduction velocity consistent with optic pathway demyelination, waveforms preserved but delayed (typical demyelinating pattern). **(Bottom Panels)**: Post-treatment VEP (Right and Left Eyes): Normalization of P100 latencies, Right eye: ~112.8 ms, Left eye: ~112.2 ms, improved waveform morphology and timing, findings consistent with remyelination and functional recovery of visual pathways.

The differential diagnosis included MOG-associated disease, multiple sclerosis, vascular brainstem syndromes, infectious rhombencephalitis, and neoplastic processes. Serological testing using a fixed-cell-based assay was strongly positive for AQP4-IgG antibodies. Cerebrospinal fluid analysis demonstrated mild lymphocytic pleocytosis (17 cells/μL with 94% lymphocytes), normal glucose levels (82 mg/dL), mildly elevated protein (37.3 mg/dL), and absent oligoclonal bands. Based on these findings and the characteristic clinical-radiological presentation, the patient fulfilled the 2015 International Panel for NMO Diagnosis (IPND) criteria for NMOSD (1).

The patient was initially treated with intravenous methylprednisolone (1 g daily for 5 days), followed by a step-down taper (500 mg for 2 days, then 250 mg for 2 days). Plasma exchange (PLEX), which is recommended for steroid-refractory NMOSD attacks, was not available at our center. Therefore, intravenous immunoglobulin (IVIg) was administered as an alternative escalation therapy at 0.4 g/kg/day for 5 days. Marked clinical improvement was achieved, with reduction of nystagmus (though still present in mid and lateral gaze), decreased hypophonia and dysphagia, and improved visual acuity. Mild residual cerebellar ataxia persisted, manifesting as slight retropulsion with leftward deviation. At discharge, her EDSS score was 5.0. She was prescribed. She was discharged on prednisolone 20 mg twice daily and scheduled for weekly neurology follow-up visits.

Following discharge, maintenance therapy with ravulizumab, a complement C5 inhibitor, was initiated 69 days after symptom onset. Prior to initiation of ravulizumab, and 1 month after IVIg infusion, the patient received appropriate meningococcal vaccination, including **Menactra (MenACWY)** administered as two doses 8 weeks apart, and **Bexsero (MenB-4C)** administered as two doses 4 weeks apart; ravulizumab therapy was commenced 2 weeks after completion of the meningococcal vaccination schedule, obviating the need for antibacterial prophylaxis. The patient received a loading dose of 2,400 mg IV (40 kg body weight), followed by maintenance dosing of 3,000 mg after 2 weeks, with subsequent infusions every 8 weeks. Adjunctive oral prednisone (20 mg daily) was gradually tapered by 5 mg every 3 weeks until complete discontinuation. The treatment regimen was well-tolerated with no infusion reactions or infections reported.

Three-month follow-up MRI demonstrated near-complete resolution of the previously seen longitudinally extensive lesion involving the dorsal-posterior medulla oblongata (area postrema) and upper cervical spinal cord, with only minimal residual signal changes remaining (Figure 3). Visual evoked potential showed normalized latencies bilaterally, indicating full recovery of optic nerve function (Figure 2). CVF was normal bilaterally. Subsequent AQP4-IgG testing 1 and 2 years after treatment initiation confirmed negative antibody status.

**Figure 3:**
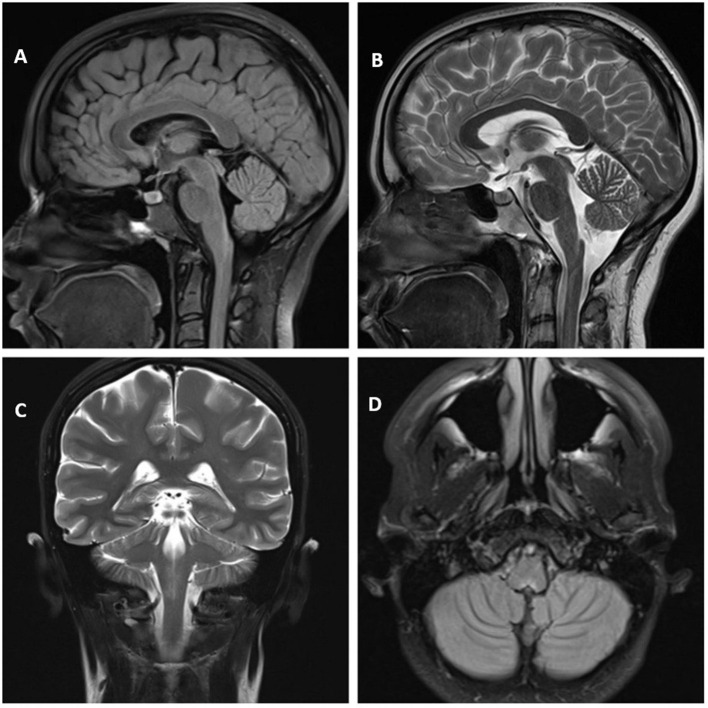
Follow-up brain MRI at 3 months after initiation of ravulizumab. **(A)** Sagittal FLAIR MRI: Near-complete resolution of the previously seen dorsal medullary (area postrema) hyperintensity. **(B)** Sagittal T2-weighted MRI: Marked reduction/disappearance of prior brainstem T2 hyperintensity and restoration of normal signal at the cervicomedullary junction. **(C)** Coronal T2-weighted MRI: Resolution of prior midline brainstem lesion. **(D)** Axial FLAIR/T2 MRI: absence of previously identified dorsal medullary lesion.

At nearly 2 years of follow-up since the initial attack, the patient continues to remain relapse-free with no new clinical attacks or radiographic evidence of disease activity. During the 2-year follow-up period, no significant adverse events or serious infections were observed. The patient tolerated ravulizumab well, with no meningococcal or other opportunistic infections reported. This sustained treatment response—among the longest reported follow-up durations for ravulizumab in NMOSD—provides real-world validation of the CHAMPION-NMOSD trial results, demonstrating durable efficacy after post-acute initiation of ravulizumab.

## 3 Discussion

This report describes a 20-year-old female with AQP4-IgG-seropositive NMOSD, presenting with a classic triad of area postrema syndrome (APS), longitudinally extensive transverse myelitis (LETM), and optic neuritis, fulfilling the 2015 IPND diagnostic criteria for NMOSD (1). The case exemplifies critical aspects of NMOSD pathophysiology, diagnosis, and evolving management paradigms.

The initial presentation with persistent nausea and vomiting led to a preliminary diagnosis of gastritis, illustrating a common diagnostic pitfall in NMOSD. APS, characterized by intractable nausea, vomiting, and hiccups, may occur in the absence of focal neurological deficits and is frequently misattributed to gastrointestinal etiologies (13). This overlap often results in delayed neurological evaluation and postponement of appropriate immunotherapy. The area postrema, a circumventricular structure with high aquaporin-4 expression and an incomplete blood–brain barrier, is particularly vulnerable in NMOSD, explaining its early involvement. Clinicians should maintain a high index of suspicion for NMOSD in patients with persistent, unexplained vomiting unresponsive to standard gastrointestinal management, particularly when initial investigations are unrevealing. Early recognition of APS is essential to reduce diagnostic delay and improve clinical outcomes (1).

Current management strategies in the UAE emphasize high-dose IV methylprednisolone (93.8%) and plasma exchange (34%) for acute attacks, with rituximab emerging as the preferred maintenance therapy (83.3% efficacy). However, access to newer biologics like satralizumab and inebilizumab remains limited (14–16).

Ravulizumab, a long-acting C5 inhibitor, prevents terminal complement activation by blocking C5 cleavage into C5a and C5b, thereby inhibiting membrane attack complex (MAC) formation and subsequent astrocyte damage in AQP4-IgG+ NMOSD (3). It demonstrates significant efficacy in AQP4-IgG-positive NMOSD, achieving a 98.6% reduction in relapse risk with zero relapses in the CHAMPION-NMOSD trial (84 patient-years of follow-up) (7). Its long-acting C5 inhibition (half-life = 50 days) enables 8-week dosing while maintaining >98% complement blockade (17).

Acute therapy led to rapid and marked clinical improvement. At the same time, maintenance treatment with ravulizumab demonstrated sustained efficacy, evidenced by the absence of relapses, a stable neurological examination, no new MRI lesions, no visual disturbances, and no progression of spinal cord atrophy over 2 years (Table 1).

**Table 1:** Longitudinal clinical, serological, and radiological outcomes.

Timepoint	AQP4-IgG status	MRI findings	Clinical status	EDSS	Relapse status
Admission	Positive	Brainstem (area postrema) lesion and a longitudinally extensive cervical cord lesion (Figure 1)	Brainstem and cerebellar syndrome with early bulbar involvement	5.0	Index attack
ICU peak	Not performed	No significant interval radiological change reported	Rapid deterioration with severe bulbar dysfunction and cerebellar syndrome requiring ICU care	8.0	Ongoing index attack
At discharge	Not performed	Not performed	Cannot tandem walk, has marked truncal ataxia, oscillopsia from nystagmus, frequent falls, and requires support	5.0	Ongoing index attack
3 months (post-ravulizumab)	Negative	Near-complete resolution of prior brainstem and cervical cord lesions (Figure 3)	Marked clinical improvement with recovery of bulbar and cerebellar function	1	No relapse
1-year follow-up	Negative	No new lesions; stable imaging with no interval changes	Neurologically stable, minimal or no residual deficit	0	No relapse
2-year follow-up	Negative	No new lesions; stable imaging with no interval changes	Sustained clinical stability	0	No relapse

AQP4-IgG, aquaporin-4 immunoglobulin G; EDSS, Expanded Disability Status Scale; MRI, magnetic resonance imaging.

The therapeutic decision-making in this case was guided by both disease biology and clinical severity. The patient had AQP4-IgG-positive NMOSD with area postrema syndrome, brainstem involvement, longitudinally extensive cervical myelitis, and rapid deterioration with bulbar symptoms and dyspnea requiring ICU transfer, all of which indicated a high-risk attack with substantial potential for early disability (1, 18). Acute treatment was initiated with high-dose intravenous methylprednisolone; however, because PLEX was not available at our center, IVIg was used as escalation therapy (4). After acute stabilization, maintenance treatment selection was informed by the complement-mediated pathobiology of AQP4-IgG-positive NMOSD, the severity of the index attack, and contemporary recommendations favoring early use of high-efficacy targeted therapy in appropriate patients. Ravulizumab was therefore selected following meningococcal vaccination, with the aim of reducing the risk of early relapse and limiting further neurological injury. This treatment choice also reflects real-world practice considerations in the UAE, where access to advanced biologics may be variable despite increasing recognition of their clinical value.

In our case, the patient experienced AQP4-IgG seroconversion (from positive to negative) following ravulizumab treatment, despite the drug's mechanism of action targeting only complement (C5) inhibition, without direct effects on antibody production. This finding suggests that seroconversion may be attributed to reduced inflammation and decreased antigenic stimulation due to effective complement blockade. AQP4-IgG testing remained persistently negative on repeated assessments, including at 2-year follow-up after initiation of ravulizumab therapy. Given the sustained seronegativity, it is less likely that this finding reflects a transient effect of acute-phase treatments such as corticosteroids or IVIg, which were administered only during the initial attack. Nevertheless, we acknowledge that ravulizumab, as a complement C5 inhibitor, is not known to suppress antibody production directly. Therefore, while the temporal association and durability of seronegativity are notable, a causal relationship cannot be definitively established. This observation should be interpreted with caution and considered hypothesis-generating, warranting further investigation.

Longer-term data on ravulizumab in AQP4-IgG-positive NMOSD are emerging. In CHAMPION-NMOSD, interim extension results reported no adjudicated on-trial relapses through a median treatment duration of 138 weeks (19), and the final ECTRIMS 2025 long-term analysis reported maintained relapse prevention through a median follow-up of 170.3 weeks. By comparison, published real-world and case-based reports have generally described shorter follow-up, including a UAE post-marketing cohort with a median follow-up of 14.5 months and a recent early-treatment case report reporting no relapses over 15 months (20, 21). Against this background, our patient's sustained clinical and radiological stability over nearly 2 years represents one of the longer published single-case follow-up observations for ravulizumab in NMOSD. However, longer follow-up is available from extension-trial datasets. A comparison of reported NMOSD cases treated with ravulizumab with the present case is shown in Table 2.

**Table 2:** Comparison of reported NMOSD cases treated with ravulizumab vs. the present case.

References	Clinical setting	Timing/reason for ravulizumab	Follow-up	Main outcome
Amano (23)	Single-center case series of 4 patients with AQP4-IgG-positive NMOSD managed in heterogeneous real-world contexts, including post-relapse treatment, switching from satralizumab, acute COVID-19, and sequential/concomitant rituximab use.	Used primarily for relapse prevention or as a bridging strategy during treatment transition; not evaluated as a standardized early rescue monotherapy approach.	9–18 months from ravulizumab initiation across the 4 cases (Patient 1: 18 months; Patient 2: 16 months; Patient 3: 9 months; Patient 4: 11 months).	No relapses were reported during the documented follow-up periods. No major safety signals were observed during 6 months of combined ravulizumab/rituximab exposure. Symptom improvement was variable, and residual neurological symptoms persisted in several cases.
Jacob et al. (24)	Single-case report of pregnancy in AQP4-IgG-positive NMOSD.	Ravulizumab was initiated shortly after conception and continued throughout pregnancy because treatment decisions were constrained by pregnancy.	Through term delivery, with infant well at 4 months of age; the maternal postpartum interval was not separately quantified in the report.	Pregnancy was uneventful, a healthy term infant was delivered, and cord-blood testing confirmed transplacental ravulizumab exposure with low C5 activity; the infant remained clinically well at 4 months.
Neundorf et al. (21)	Single-case report of AQP4-IgG-positive NMOSD in a 59-year-old woman with severe LETM (C2–T5) and associated interstitial pneumonitis.	Ravulizumab was started 13 days after attack onset in conjunction with prior acute therapy, including high-dose methylprednisolone and immunoadsorption.	15 months after ravulizumab initiation.	Follow-up showed no relapses, reduction in spinal cord edema, partial resolution of pulmonary infiltrates, and neurological improvement; however, substantial residual disability persisted.
Valdés et al. (8)	Single-case report of a fulminant, treatment-refractory AQP4-IgG-positive NMOSD attack with bilateral corticospinal tract involvement and asymmetrical tetraparesis.	Ravulizumab was administered on day 12 of the attack after continued deterioration despite intravenous methylprednisolone and plasma exchange; it was used as a salvage/stabilization intervention.	Short-term in-hospital follow-up only: discharge on day 19 of hospitalization (7 days after ravulizumab initiation).	Neurological deterioration ceased after ravulizumab administration, rehabilitation progressed, and the patient was discharged with improved strength and function; however, significant disability remained at discharge (EDSS 7.0).
Jacob et al. (20)	Multicenter retrospective post-marketing cohort study from 2 major UAE neurological centers including 9 patients with AQP4-IgG-positive NMOSD.	Ravulizumab was used in routine clinical practice for relapse prevention in a real-world post-marketing setting; 44.4% of patients were treatment naïve.	Median 14.5 months (IQR 6.25).	All patients remained relapse-free during follow-up; annualized relapse rate fell from 0.9 ± 0.5 to 0, mean EDSS improved from 3.9 ± 2.4 to 2.2 ± 3.0, and no meningitis cases were observed. One patient discontinued treatment because of insurance denial.
Present case	Severe AQP4-IgG-positive NMOSD with brainstem/area postrema involvement, LETM, bulbar dysfunction, ICU deterioration, and abnormal baseline VEP.	Post-acute initiation of ravulizumab 69 days after symptom onset.	Approximately 24 months from the index attack (reported in the draft as “nearly 2 years”).	AQP4-IgG became negative by 3 months and remained negative at 1 and 2 years; MRI showed marked lesion resolution by 3 months with no interval disease activity at 1 and 2 years; VEP improved/normalized; no relapses were observed during follow-up.

AQP4, aquaporin 4; ICU, intensive care unit; IgG, immunoglobulin G; LETM, longitudinally extensive transverse myelitis; MRI, magnetic resonance imaging; NMOSD, neuromyelitis optica spectrum disorder; VEP, visual evoked potential.

This therapeutic advance is critical given that untreated NMOSD is associated with rapid disability accumulation (58% risk of permanent disability within 1 year) (12) and increased mortality risk per attack (22). Post-acute initiation of ravulizumab is recommended to prevent relapses in AQP4-IgG-positive NMOSD, based on its demonstrated efficacy and on consensus recommendations to start high-efficacy maintenance therapy promptly after diagnosis (4, 7, 14). The CHAMPION-NMOSD trial confirmed ravulizumab efficacy as monotherapy (51.7% of patients) in AQP4-IgG-positive NMOSD patients with a manageable safety profile, though meningococcal vaccination remains essential (7).

This report has several limitations. First, it describes a single patient and therefore cannot establish treatment efficacy, comparative effectiveness, or generalizability. Second, although the patient remained relapse-free for nearly 2 years, longitudinal follow-up was not documented with the same granularity at all time points, and standardized intermediate outcome measures were not uniformly available throughout routine care. Third, the observed AQP4-IgG seroconversion after ravulizumab should be interpreted cautiously, as ravulizumab is not known to directly suppress antibody production, and a causal relationship cannot be inferred from a single case. Finally, the favorable outcome observed here likely reflects the combined effect of acute-phase therapy, supportive care, and subsequent maintenance complement inhibition, making it difficult to isolate the specific contribution of any one intervention.

## 4 Conclusion

This case demonstrates the diagnostic and therapeutic challenges of AQP4-IgG-positive NMOSD in a young patient presenting with area postrema syndrome. The patient remained clinically stable and free of new MRI activity through ~24 months of follow-up on ravulizumab. The case highlights the importance of early recognition and treatment to prevent disability, while confirming the efficacy of complement inhibition therapy.

## Data Availability

The datasets presented in this article are not readily available because of ethical and privacy restrictions. Requests to access the datasets should be directed to the corresponding author.
